# Regulation of Hypoxia-Induced Cell Death in Human Tenocytes

**DOI:** 10.1155/2012/984950

**Published:** 2012-12-06

**Authors:** Min Liang, Hannah R. Cornell, Nasim Zargar Baboldashti, Mark S. Thompson, Andrew J. Carr, Philippa A. Hulley

**Affiliations:** ^1^Botnar Research Centre, Nuffield Department of Orthopaedics, Rheumatology and Musculoskeletal Sciences, University of Oxford, Oxford OX3 7LD, UK; ^2^Department of Periodontology, Guanghua School of Stomatology and Hospital of Stomatology, Sun Yat-sen University, 56 Lingyuan Road West, Guangzhou 510055, China; ^3^Institute of Biomedical Engineering, University of Oxford, Old Road Research Campus, Oxford OX3 7DQ, UK; ^4^The Nuffield Department of Orthopaedics, Rheumatology and Musculoskeletal Sciences (NDORMS), NIHR Musculoskeletal BRU, University of Oxford, Oxford OX3 7LD, UK

## Abstract

Degenerate shoulder tendons display evidence of hypoxia. However tendons are relatively avascular and not considered to have high oxygen requirements and the vulnerability of tendon cells to hypoxia is unclear. Cultured human tenocytes were exposed to hypoxia and the cellular response detected using QPCR, Western blotting, viability, and ELISA assays. We find that tenocytes respond to hypoxia *in vitro* by activating classical HIF-1**α**-driven pathways. Total hypoxia caused significant tenocyte apoptosis. Transcription factors typically involved in hypoxic response, HIF-1**α** and FOXO3A, were upregulated. Hypoxia caused sustained upregulation of several proapoptotic proteins known to mediate hypoxia-induced apoptosis, such as Bnip3 and Nix, but others were unchanged although they were reportedly hypoxia-sensitive in other cell types. Antiapoptotic proteins Bcl2 and Bcl-xL were unchanged by hypoxia. Normal human tenocytes expressed all isoforms of the hypoxia-induced vascular growth factor VEGF except VEGF-D. Hypoxia markedly upregulated VEGF-A mRNA, followed by increased VEGF protein secretion. However treatment with VEGF did not improve tenocyte survival. As a protective strategy for tenocytes at risk of hypoxic death we added prosurvival growth factors insulin or platelet rich plasma (PRP). Both agents strongly protected tenocytes from hypoxia-induced death over 48 h, suggesting possible efficacy in the acute postrupture tendon or integrating graft.

## 1. Introduction

The tissue-specific physiology of tendon is adjusted to extreme mechanical loading, which results in acute and repetitive reductions in blood perfusion and therefore a likely ability to tolerate transient hypoxia. With increasing age tendons are also at increasing risk of degeneration and tear or rupture with the inevitable disruption of blood supply. Tendons such as the rotator cuff in the shoulder are prone to a progressive degenerative process leading to tears in as many as 54% of the population aged above 60 [1]. These injuries are disabling, painful, and heal poorly. In most cases, the causes of tendon weakness and degeneration are not known and in particular the role of hypoxic insults is not understood.

The enthesis is poorly vascularised in all tendons as is the so-called critical zone, just proximal to the tendon/bone attachment, where the majority of rotator cuff tears take place [2–4]. However, tendon itself is not without blood vessels and there is evidence that considerable vascular remodeling takes place during tendon healing. The vessel count in early stage rotator cuff tears increases by up to 4-fold in stark contrast with end-stage degenerative cuff tears which have lower vessel counts than uninjured tendon [5, 6]. Despite its relatively avascular appearance, tendon is more oxygen-dependent than other joint tissues such as cartilage and requires enhanced perfusion during repair [7].

Metabolically tendon is described as reliant on oxidative phosphorylation during development and early growth with a shift towards anaerobic glycolysis as the tissue matures [7]. Following injury tenocytes become more metabolically active with a marked increase in oxidative phosphorylation during the repair process [7]. Given this ability to adapt to changing metabolic demands and the low energy requirements of mature tenocytes it is sometimes assumed that tendon is resistant to changes in blood supply and oxygen level.

Recent studies have however found evidence of classical hypoxia response pathways in tendon [8, 9]. Torn rotator cuffs express high levels of HIF-1alpha (HIF-1*α*) as well as at least two HIF-driven genes, vascular endothelial growth factor (VEGF) [6] and the proapoptotic Bcl2 family member Bnip3 [8, 9]. The forkhead transcription factor FOXO3A has also been reported to be upregulated in response to hypoxia [10] and to strongly regulate Bnip3 [11]. Expression of Bnip3 precedes and correlates with increasing levels of apoptotic cell death in advancing stages of rotator cuff degeneration [8]. These data suggest that hypoxia may be a relevant damage factor in tendon injury and that appropriate vascular response may be essential for normal repair and remodeling. However the expression of VEGF is also increased by inflammation or mechanical load [12] and Bnip3 is not exclusively regulated by hypoxia [11, 13]. It is not clear from these studies what role hypoxia plays in driving this response or how involved the tenocytes themselves are rather than surrounding tissue such as bursa or blood vessels. 

Exogenous growth factors are potentially useful agents for the enhancement of tendon healing. VEGF and IGF have been reported to protect cells such as neurons from hypoxic insult [14, 15] and IGF has also been reported to protect tenocytes against hypoxia [16]. Similarly second messengers downstream of growth factor receptors such as cAMP can mimic this prosurvival effect [17–19]. Platelet rich plasma (PRP) is currently undergoing investigation as an autologous growth factor therapy in many tissues including tendon [20]. Platelet rich plasma is, by definition, a volume fraction of the plasma, having a platelet concentration above baseline (whole blood) [21]. Platelets are active participants in different healing processes in the body [22]. Platelet *α*-granules are rich in growth factors and other active molecules that have significant roles in facilitating healing. Growth factors released by platelets upon platelet activation include platelet-derived growth factor (PDGF), endothelial growth factor (EGF), insulin-like growth factor (IGF-I), transforming growth factor-*β*I (TGF-*β*I), and vascular endothelial growth factor (VEGF) [22, 23]. These growth factors and many other proteins secreted by platelets are active in the healing cascade by promoting cell proliferation and migration, synthesis of extracellular matrix proteins such as collagen and supporting angiogenesis and vascularisation. 

PRP has shown activity in tenocytes and healing tendons although no studies have been performed to establish efficacy against hypoxic damage. Schnabel et al., have found an increase in collagen type I gene expression in cultured tendon cells treated with PRP [24]. In an animal study, greater maturation in tendon callus has been reported after using PRP for augmentation of rat Achilles tendon tears. The same study has also reported higher ultimate stress and increased force to failure in PRP-treated animal tendons [25]. In a recent study, the mobilization of circulation-derived cells was enhanced in the area of PRP injection. They also reported an increase in the production of type I collagen and macrophage proliferation at 3 and 7 days [26]. However direct prosurvival effects of PRP have not yet been demonstrated on tenocytes and we have therefore compared PRP to insulin, VEGF and cAMP in treatment of hypoxic human tenocytes.

We hypothesize that tenocytes are vulnerable to hypoxia-induced cell death and that growth factors may offer a degree of protection, in line with effects on other cell types. We have therefore set out to characterize the response of normal human tenocytes to acute hypoxia (0.1% oxygen) in both high and low serum cultures, to assess the capacity of tenocytes themselves to release proangiogenic factors and to devise protective strategies to enhance tenocyte survival after ischemic insult.

## 2. Materials and Methods

All chemicals and reagents were obtained from Sigma (Poole, UK) except where otherwise stated. Whole blood was obtained from healthy volunteers and surplus human hamstring tendon tissue was obtained during anterior cruciate ligament reconstruction via the Oxford Musculoskeletal Biobank with informed consent and in full compliance with UK HTA and MREC requirements.

### 2.1. Cell Culture

Primary human tenocytes were derived from healthy hamstring tendon explants, collected fresh from surgery. The cells were grown in bicarbonate-buffered DMEM: F12 (Cambrex, Wokingham, UK) with 10% heat-inactivated FCS (Biosera, Ringmer, UK), 100 U/mL penicillin, and 100 mg/mL streptomycin. For hypoxia experiments, the cells were cultured in 10% or 1% FCS-containing medium with 0.1% oxygen for the times indicated. The cells cultured in 10% FCS with atmospheric oxygen served as control. Cells within three passages were used for all experiments except initial phenotyping experiments which were run out to 8 passages. Human myeloma cell line RPMI 8226 cells were cultured in RPMI medium and used as positive control for Bcl2 family protein expression and VEGF gene expression. Recombinant human VEGF B 167 [14] was obtained from R&D systems (Abingdon, UK).

### 2.2. Western Blotting

Total cell protein was extracted from human tenocytes within three passages. Protein concentration was measured using the BCA protein assay kit (Pierce Biotechnology, Rockford, IL). Equal protein samples were separated on SDS-PAGE gels by electrophoresis and transferred onto a polyvinylidene fluoride (PVDF) membrane (Millipore, Bedford MA). Prestained Precision Plus molecular weight markers (BioRad, Hercules, CA) were used to estimate molecular weight. Rabbit polyclonal primary antibodies: anti-Bim and anti-cleaved Caspase-3 antibodies were purchased from Calbiochem (San Diego, CA); anti-Bax, anti-Bad, Puma, Bmf, Noxa antibodies were from Cell Signalling Technologies (Beverley, MA); anti-Bak antibody was from Upstate (Dundee, UK); Bcl2, Bcl-xL (Santa Cruz, CA), Nix (Axxora, Lausen, Switzerland). Mouse monoclonal primary antibodies: Bnip3 (Axxora, Lausen, Switzerland), HIF-1*α* (BD Biosciences, Erembodegem, Belgium), *β*-Tubulin (Sigma, Poole, UK), FOXO-3A (Epitomics, Burlingame, CA). Secondary antibodies were goat anti-rabbit horseradish-peroxidase conjugated antibody (Abcam, Cambridge, UK) and goat anti-mouse horseradish-peroxidase conjugated antibody (Pierce Biotechnology, Rockford, IL). Immunocomplexes were detected using enhanced chemiluminescence detection system or Supersignal West Dura extended duration substrate (Pierce Biotechnology, Rockford, IL) and signals were captured using a UVP Chemidoc-it. VisionWorksLS software v6.7.4 was used to measure the density of bands of interest. *β*-Tubulin was stable under hypoxic conditions and served as internal control.

### 2.3. Standard RT-PCR and Real-Time Quantitative RT-PCR

Total mRNA was isolated from cells using RNeasy Mini kits (Qiagen, GmbH, Hilden, Germany), according to the manufacturer's protocol. RNase-Free DNase Set (Qiagen, GmbH, Hilden, Germany) was used to remove genomic DNA contamination. 0.3 *μ*g total RNA in a 20 *μ*L reaction volume was reverse-transcribed using SuperScript II Reverse Transcriptase (Invitrogen, Paisley, UK) in the presence of oligo-dT primer (Invitrogen, Paisley, UK). Samples without reverse transcriptase served as negative controls in real-time PCR reactions to exclude genomic DNA contamination. No-template control was always included to indicate that there was no contamination in real-time PCR reagents. Scleraxis and *β*-actin primers were Quantitect primers from Qiagen and other primers [27, 28] were from MWG Biotech (London, UK). Human VEGF-A primers to detect all splice variants, forward 5′-CTTGCCTTGCTGCTCTACC-3′ (Exon 1) and reverse 5′-CACACAGGATGGCTTGAAG-3′ (Exon 3); VEGF-A primers to distinguish different splice variants, forward 5′-CTCACCAAGGCCAGCACATAGG-3′ (Exon 4), reverse 5′-ATCTGGTTCCCGAAACCCTGAG-3′ (Exon 8). Human VEGF-B forward primer was 5′-TGTGTATACTCGCGCTACCTG-3′ and reverse primer was 5′-CATTCACACTGGCTGTGTTC-3′. Human VEGF-C forward primer was 5′-GTCTGTGTCCAGTGTAGATG-3′ and reverse primer was 5′-AGGTAGCTCGTGCTGGTGTT-3′. Human VEGF-D forward primer was 5′-CAGTGAAGCGATCATCTCAGTC-3′ and reverse primer was 5′-TACGAGGTGCTGGTGTTCATAC-3′. Standard PCR reactions were performed using a Thermo Hybaid system. The amplicons were separated by electrophoresis to confirm the size of the products. Real-time quantitative PCR reactions were performed using a Corbett Rotor-Gene 3000 using QuantiTect SYBR Green PCR kit (Qiagen, GmbH, Hilden, Germany). Samples were run in duplicate and the average value was used for analysis. Comparative Quantitation analysis [29, 30] (Corbett Rotor-Gene version 6.0.1) was used to compare the gene expression of VEGF-A in treated samples relative to control cells and normalised to the housekeeping gene *β*-actin. 

### 2.4. ELISA

Cell culture supernatants were collected from hypoxia treated samples or control samples and kept at −80°C until use. VEGF-A expression was measured by using Quantikine Human VEGF ELISA kit, according to the manufacturer's protocol (R&D Systems, Abingdon, UK). Absorbance was measured by microplate reader at 450 nm. Results were normalized to total protein of the cells and data were presented as *μ*g VEGF-A/*μ*g total protein.

### 2.5. Apoptosis Determination

An *in situ* cell death detection kit (Roche Applied Science, Penzberg, Germany) was used to perform the TUNEL reaction to identify apoptotic cells with fragmented DNA, according to the manufacturer's instructions. DAPI was used to visualise all nuclei. The cells were observed and photographed using an Olympus BX40 microscope and Olympus DP70 camera. TUNEL-and DAPI-positive cells were counted manually using cell^*∧*^F program (Olympus, Soft Imaging System GmbH, Münster, Germany) and 15–20 fields were taken from each slide. 

### 2.6. Platelet Rich Plasma Treatment

Platelet Rich Plasma was generated from fresh whole human blood using Smith & Nephew's Caption device. 55 mL from a healthy volunteer was collected directly into a syringe containing 5 mL acid citrate dextran anticoagulant. Platelets were concentrated according to Smith & Nephew's protocol and stored frozen until use (freezing was found to reduce platelet activity and should be avoided where possible). 1 mL of PRP was activated with bovine thrombin and the resulting clots left to condition 9 mL of standard culture medium containing 1% FCS for 16 h. Conditioned medium was centrifuged at 1400 g and syringe-filtered to remove debris prior to use. Hamstring tenocytes were cultured in 10% FCS until 70% confluence when medium was replaced with 1% FCS. Paired cultures were treated with or without 10% PRP conditioned medium and placed either in a multigas incubator set at 0.1% oxygen or under standard culture conditions of atmospheric oxygen for 48 h. Cells were stained using Live & Dead stain (Invitrogen, Paisley, UK) and imaged with an Olympus BX40 microscope and Olympus DP70 camera, followed by analysis with ImageJ software. 

### 2.7. Statistical Analysis

Data are presented as means ± SD or SEM from three or more separate experiments, as indicated. Data were analyzed using GraphPad Prism v 4.03 (GraphPad Software, Inc., La Jolla, CA). Student's *t*-test (for single comparison) or one-way ANOVA (for multigroup comparisons) with either Dunnett or Tukey post hoc test were used as appropriate. *P* < 0.05 was regarded as denoting statistical significance.

## 3. Results

### 3.1. Characterisation of Normal Human Hamstring Tenocytes *In Vitro*

**Figure 1:**
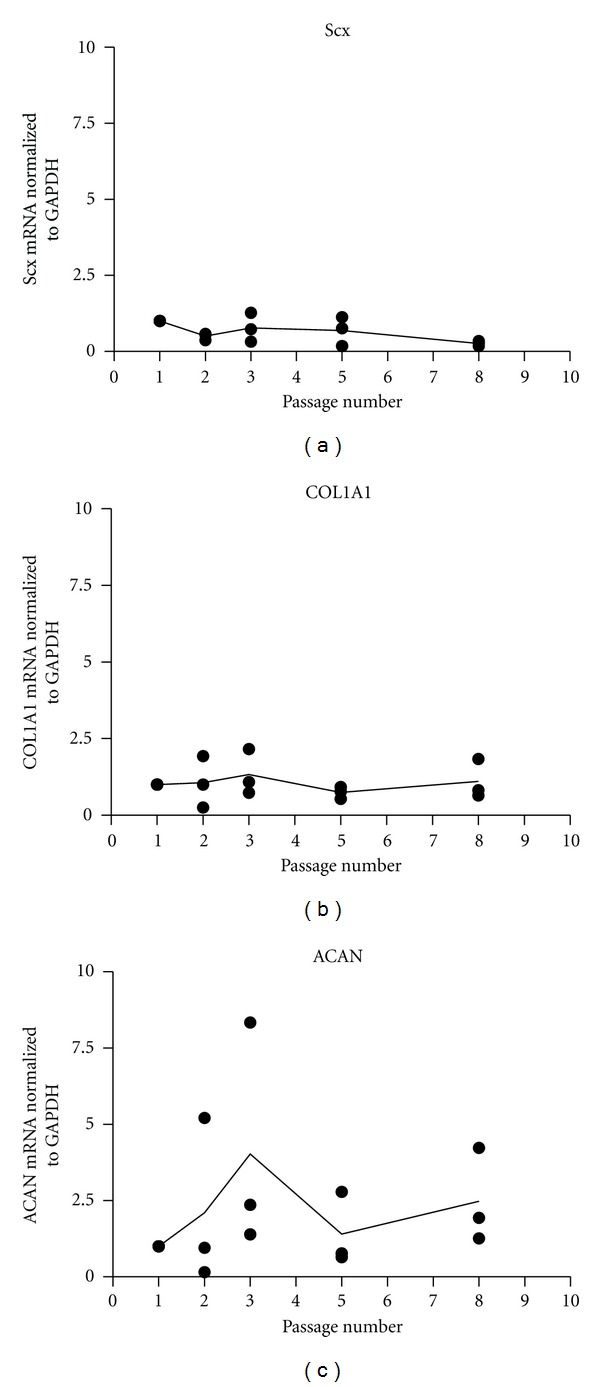
Human tenocytes express stable levels of scleraxis, collagen and aggrecan until passage 5 but scleraxis drops between passage 5 and 8. Human tenocytes from 3 individual donors were cultured in 10% FCS-containing medium under normoxia. (a) the expression of tenocyte ACAN, COL1A1, and Scx was quantified by rtq-PCR with *β*-actin as house-keeper.

We have established a protocol for deriving large numbers of healthy human tenocytes from hamstring tendon. Hamstring tendon is used during routine anterior cruciate ligament reconstruction and the discarded stubs are collected from theatre, finely diced and explanted in tissue culture. Over a period of 2 weeks tenocytes migrate out of the explanted tissue pieces and can be amplified in culture. To assess phenotypic drift in our cultures mRNA was collected from tenocytes derived from 3 donors at passage 1, 2, 3, 5, and 8 and expression of collagen I, aggrecan core protein and the tenocyte transcription factor, scleraxis, were quantified (Figure 1). Although collagen I and aggrecan expression was relatively consistent, scleraxis levels dropped between passage 5 and 8 and therefore we have performed all tenocyte experiments on cells up to passage 3.

### 3.2. Hypoxia Rapidly Induces HIF-1*α* Expression in Human Tenocytes

**Figure 2:**
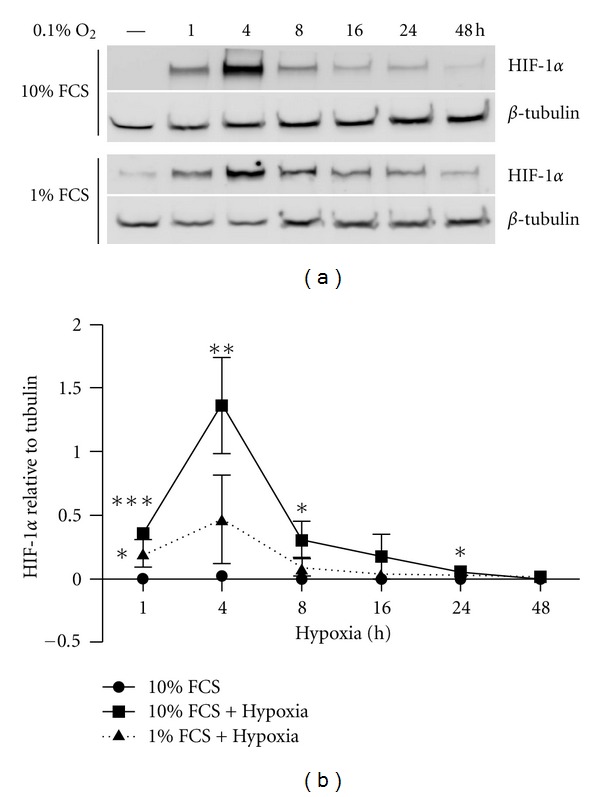
Hypoxia rapidly induces HIF-1*α* expression in human tenocytes. Primary human tenocytes were cultured in DMEM/F12 culture medium containing 10% FCS under normoxic conditions until the cells reached 70% confluence. The cells were exposed to total hypoxia (0.1% O_2_) for 1, 4, 8, 16, 24, and 48 h. (a) Representative Western blot for HIF-1*α* in tenocytes. (b) HIF-1*α* expression qualified by densitometry is presented as mean ± SE. (*n* = 3). *P < 0.05 versus normoxic control; **P < 0.01 versus normoxic control; ***P < 0.001 versus normoxic control.

To assess the response of human tenocytes to reduced oxygen, primary tenocytes were cultured in medium containing 10% FCS under normoxic conditions until the cells reached 70% confluence. Medium was then changed to either 10% or 1% FCS and the cells were exposed to total hypoxia (0.1% O_2_) for 1, 4, 8, 16, 24, and 48 h. The appropriate level of serum for primary tenocyte culture is unknown and since growth factors can protect against ischemia we compared the effect of hypoxia against a background of either high (10%) or low (1%) serum throughout this study. The cell lysates were prepared in HIF buffer [31]. Hypoxia inducible factor-1*α* subunit (HIF-1*α*) was detected by Western blotting. HIF-1*α* expression was below detectable levels under normal oxygen conditions in human tenocytes (Figure 2(a)) but accumulated rapidly with hypoxia (0.1% O_2_) in either 10% or 1% FCS containing medium (Figures 2(a) and 2(b)). HIF-1*α* reached a maximal level between 1 and 8 h. *β*-Tubulin protein is stable under hypoxic conditions and was used as internal control.

### 3.3. Human Tenocytes Express VEGF-A, B, and C but Not D mRNA

**Figure 3:**
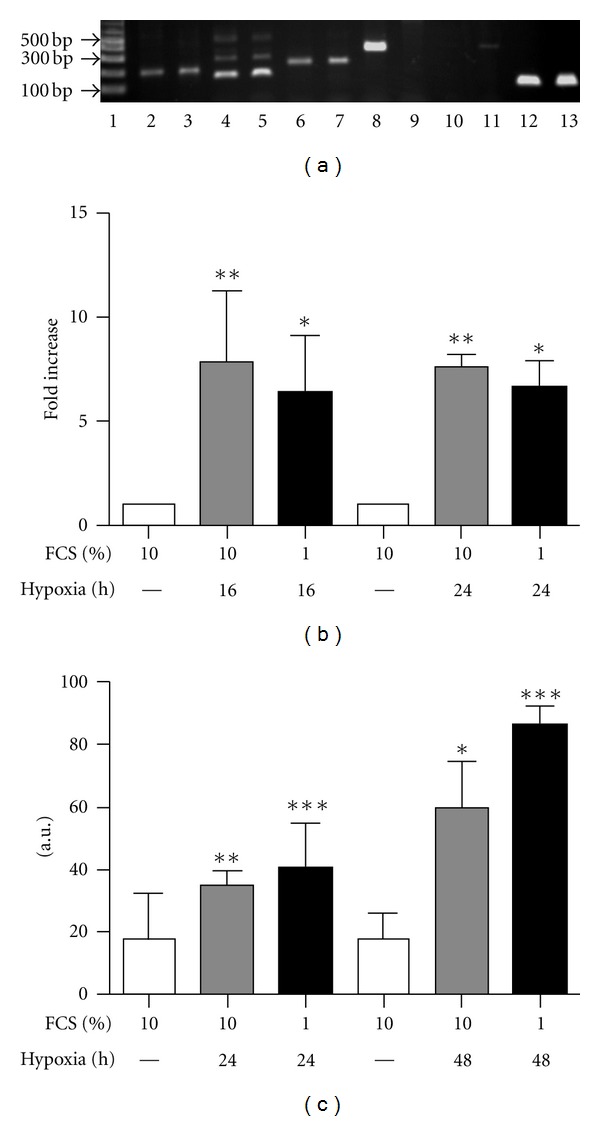
Human tenocytes express VEGF-A, B, and C but not D mRNA and hypoxia treatment upregulates VEGF-A mRNA and protein expression. (a) Gene expression of VEGF-A (lane 2 and 3), B (lane 6 and 7), C (lane 8 and 9), D (lane 10 and 11), and VEGF-A splice variants (lane 4 and 5) was determined by standard RT-PCR with 35 cycles from human tenocytes (lane 2, 4, 6, 8, 10, and 12) at passage 1 or human myeloma cell RPMI (lane 3, 5, 7, 9, 11, and 13). Products were separated by electrophoresis. *β*-actin (lane 12 and 13) was used as housekeeping gene. (b) Hypoxia upregulates VEGF-A gene expression. Real-time quantitative PCR was used to compare the relative gene expression of VEGF-A 16 and 24 h after hypoxia and data are represented as fold change (mean ± SD, *n* = 3; *P < 0.05 or **P < 0.01 versus relevant time controls). (c) Hypoxia upregulates VEGF protein secretion. Cell culture supernatants were collected after hypoxia treatment for 24 h or 48 h and ELISA was used to measure VEGF-A secretion from human tenocytes. Data are represented as *μ*g VEGF-A per *μ*g total protein (mean ± SD, *n* = 3; *P < 0.05, **P < 0.01, ***P < 0.001 versus relevant time control).

VEGF is classically upregulated in oxygen-starved tissue to restore the normal vasculature. To establish the VEGF profile of human tenocytes, total RNA was extracted from the tenocytes at passage 1 and gene expression of VEGF-A, B, C, D, and *β*-actin were determined by standard RT-PCR (Figure 3(a)). Human myeloma cell line RPMI was used as a positive control. Human tenocytes expressed VEGF-A (lane 2), B (lane 6), and C (lane 8) but not D (lane 10) mRNA. RPMI cells expressed VEGF-A (lane 3), B (lane 7), and D (lane 11) but not C (lane 9) mRNA. *β*-actin was used as internal control for tenocytes (lane 12) and RPMI (lane 13). Different VEGF-A splice variants were also detected using a primer pair that can separate the variants according to their different lengths. Both tenocytes (lane 4) and RPMI cells (lane 5) expressed four VEGF-A variants, including VEGF-121 (159 bp), 165 (291 bp), 189 (363 bp), and 206 (414 bp).

### 3.4. Both VEGF-A Gene and Protein Expression Are Upregulated by Hypoxia in Tenocytes

Following hypoxic exposure, relative VEGF-A gene expression was measured by real-time RT-PCR Figure 3(b)). VEGF-A gene expression was induced by hypoxia and reached a plateau before 24 h. VEGF-A protein release was measured by ELISA by collecting the cell culture supernatants (Figure 3(c)). The VEGF-A antibody used recognizes all VEGF-A isoforms and the results were normalized to total protein of the cells. VEGF-A release in the culture medium increased 24 h after hypoxia treatment and further accumulated in the culture medium 48 h after hypoxia when the cells were cultured in either 10% or 1% FCS containing medium.

### 3.5. Bnip3 Is Upregulated by Hypoxia in Human Tenocytes

**Figure 4:**
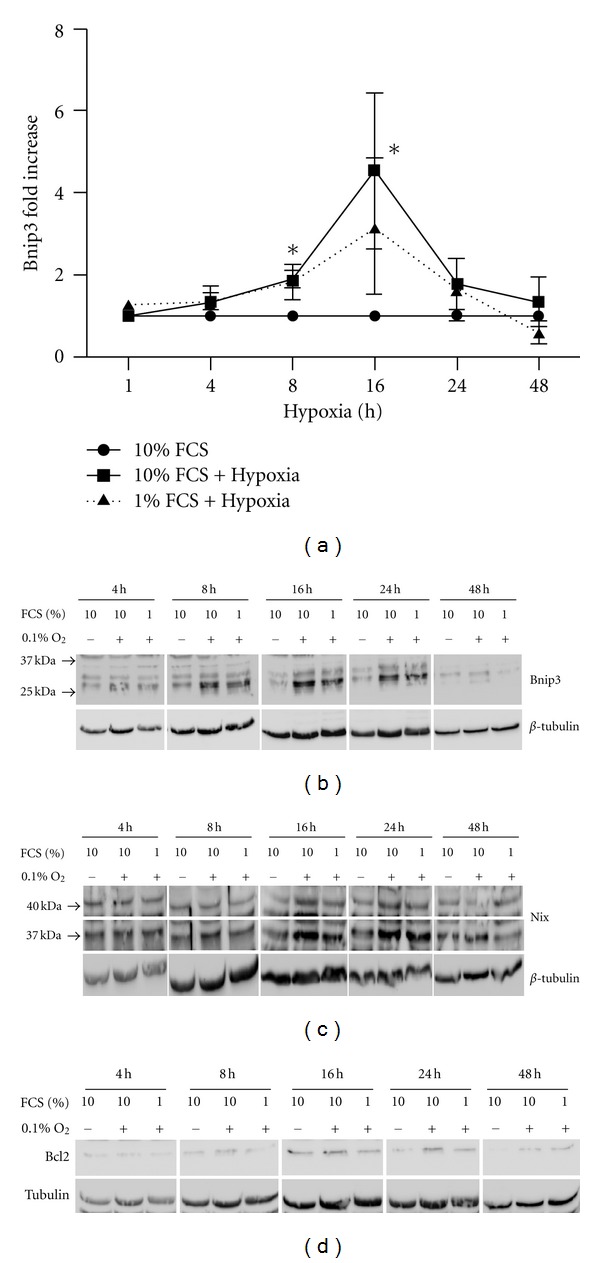
Bnip3 and Nix are upregulated by hypoxia in human tenocytes but Bcl2 is not. Primary human tenocytes were exposed to total hypoxia (0.1% O_2_) for 1, 4, 8, 16, 24, and 48 h. The cells were harvested in cell lysis buffer. (a) Representative Western blot for Bnip3 in tenocytes. (b) Fold change of Bnip3 is presented as mean ± SE (*n* = 3). *Represents *P* < 0.05 versus control. (c) Representative western blot for Nix after hypoxia treatment for 1, 4, 8, 16, 24, and 48 h in tenocytes. (d) Antiapoptotic protein Bcl2 remains at low level after hypoxia treatment. Representative Western blot for Bcl2 after hypoxia treatment for 1, 4, 8, 16, 24, and 48 h in tenocytes.

Another HIF-1alpha target gene is the proapoptotic Bcl2 family member, Bnip3 [11, 13]. Primary human tenocytes were exposed to total hypoxia (0.1% O_2_) for 1, 4, 8, 16, 24, and 48 h. The cells were harvested in standard lysis buffer. Bnip3 is a 21.5 kDa protein that typically migrates at either 35 kDa (monomer) or 60 kD (dimer) in SDS PAGE gels [32]. Multiple immuno-positive bands were recognized by Bnip3 antibody (Figure 4(a)), correlating with different Bnip3 isoforms [32]. Bnip3 expression was significantly increased at 8 h and 16 h after hypoxia treatment, slightly later than HIF-1*α* induction (Figure 4(b)) and reached a peak between 8 and 24 h in human tenocytes (Figures 4(a) and 4(b)).

Nix is a HIF-responsive Bnip3 homologue and was also found to be expressed in primary human tenocytes as a doublet at 37 kD and 40 kD [13, 33]. Nix (37 kD band) tended to increase at 16 h and 24 h after hypoxia, although there was no statistical significance (Figure 4(c)). Other proapoptotic Bcl2 family members Bim, Bak, Bax, and Puma were expressed in human tenocytes but remained unchanged throughout 48 h of hypoxia (data not shown). Noxa and Bmf expression was below detectable levels in human tenocytes but both were detected in the human myeloma cell line RPMI. Bad was also expressed in tenocytes. However, Bad expression was upregulated only by a combination of hypoxia with low serum in two out of three experiments, suggesting complex regulation (data not shown).

### 3.6. Antiapoptotic Proteins Bcl2 and Bcl-xL Remain at a Low Level after Hypoxia

The expression and regulation of antiapoptotic proteins Bcl2 and Bcl-xL were determined by Western blotting after hypoxia. Both Bcl2 (Figure 4(d)) and Bcl-xL (not shown) were expressed at low levels in tenocytes and remained unchanged after 48 h hypoxia. 

### 3.7. Insulin Protects Tenocytes from Apoptosis Induced by Hypoxia

**Figure 5:**
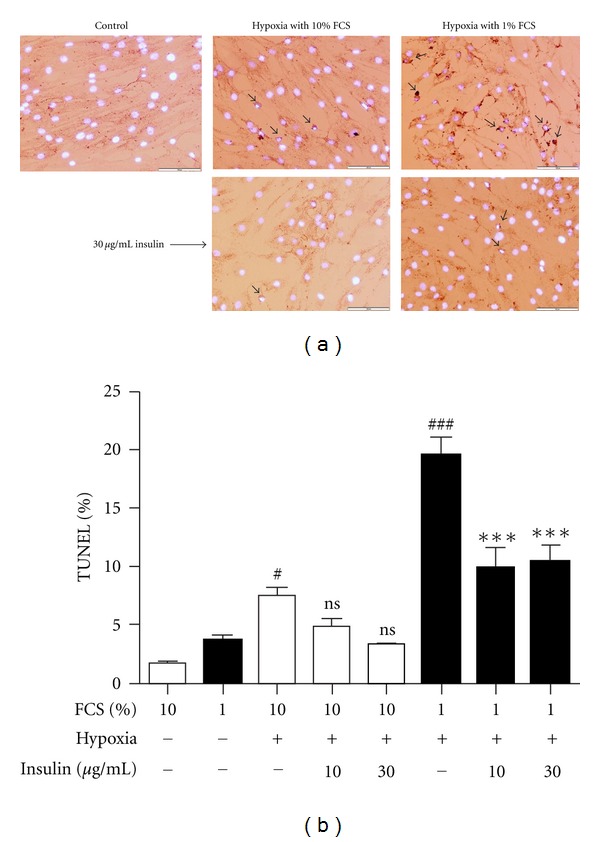
Insulin protects tenocytes from apoptosis induced by hypoxia. (a) TUNEL and DAPI double-staining from cells under normoxic control conditions (top left panel), hypoxia (0.1% O_2_) treatment with 10% FCS for 48 h (top middle panel), hypoxia treatment with 1% FCS for 48 h (top right panel) and 30 *μ*g/mL insulin treatment for 48 h with hypoxia in the presence of 10% FCS (bottom left panel) or 1% FCS (bottom right panel). (b) Percentage of apoptotic cells versus total cells (mean ± SE, *n* = 3; ^#^ *P* < 0.05 versus 10% FCS alone; ^###^ *P* < 0.001 versus 1% FCS alone; ns not significant versus 10% FCS + hypoxia; ***P < 0.001 versus 1% FCS + hypoxia).

TUNEL and DAPI double-staining of cells grown on coverslips was used to evaluate apoptosis induced by hypoxia. Under normoxic conditions with 10% FCS culture medium, tenocytes appeared healthy and spindle shaped (Figure 5(a), top left panel). Few TUNEL positive cells were observed, and DAPI staining showed normal morphology of nuclei. Serum starvation (1% FCS) slightly increased TUNEL positive cells. However, hypoxia (0.1% O_2_) significantly induced cell death from 1.6% under normoxia to 7.5% under hypoxia 48 h after treatment in human tenocytes (Figure 5(a), top middle panel and Figure 5(b)). Hypoxia combined with serum reduction (1% FCS) further increased apoptosis by 19.5% after 48 h treatment (Figure 5(a), top right panel and Figure 5(b)). Since Akt/PKB activating growth factors are strongly antiapoptotic in many cell types we used high-dose insulin to block hypoxia-induced apoptosis. At 10 *μ*g/mL or 30 *μ*g/mL insulin treatment partially protected tenocytes from apoptosis in the presence of 10% FCS (Figure 5(a), bottom left panel and Figure 5(b)) or 1% FCS (Figure 5(a), bottom right panel and Figure 5(b)). In contrast, neither recombinant VEGF-B (10–100 ng/mL) nor the stable, cell permeable cAMP analogue (8-(4-Chlorophenyl)thio-cyclic AMP; 10–100 *μ*M) demonstrated any protection (results not shown).

### 3.8. Insulin Does Not Decrease HIF1*α* Expression or Alter FOXO3A Expression

**Figure 6:**
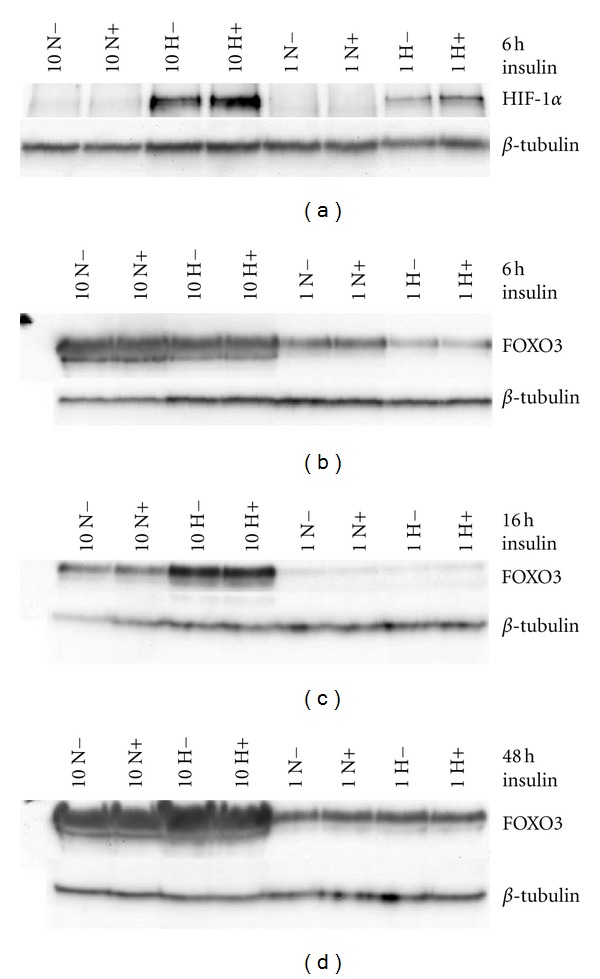
Cotreatment with insulin does not change the induction of HIF-1*α* or FOXO-3A by hypoxia. Tenocytes subjected to 0.1% oxygen upregulated HIF-1 at 6 h in 10% FCS and less strongly in 1% FCS. Cotreatment with 30 ug/mL insulin did not prevent the induction of HIF-1*α* (a). The stress responsive transcription factor FOXO3A was not changed after 6 h of hypoxia (b) but was upregulated at the later time points of 16 h (c) and 48 h (d). Cotreatment with 30 ug/mL insulin had no effect on FOXO-3A protein levels at any time-point. Representative Western blots are shown from each time-point. Experiments were repeated with cells derived from 2 donors.

In order to examine the antiapoptotic mechanism by which insulin protects hypoxic tenocytes we assessed the levels of HIF1*α* under normoxia and hypoxia. HIF1*α* protein levels were not decreased by insulin treatment at 6 h (Figure 6(a)). The forkhead transcription factor FOXO3A has been reported to be upregulated in response to hypoxia [10], to strongly regulate Bnip3 [11] and to mediate damage responses such as autophagy [11, 34]. We found that total hypoxia upregulated FOXO3A levels by 16 h in 10% but not 1% FCS (Figure 6(c)) and levels were still elevated after 48 h (Figure 6(d)). However, although insulin and other PKB activators have been described to oppose the actions of forkhead proteins we detected no decrease in FOXO3A protein levels with insulin and hypoxia cotreatment (Figures 6(b), 6(c), and 6(d)). 

### 3.9. Platelet Rich Plasma Protects Tenocytes from Hypoxia-Induced Cell Death

**Figure 7:**
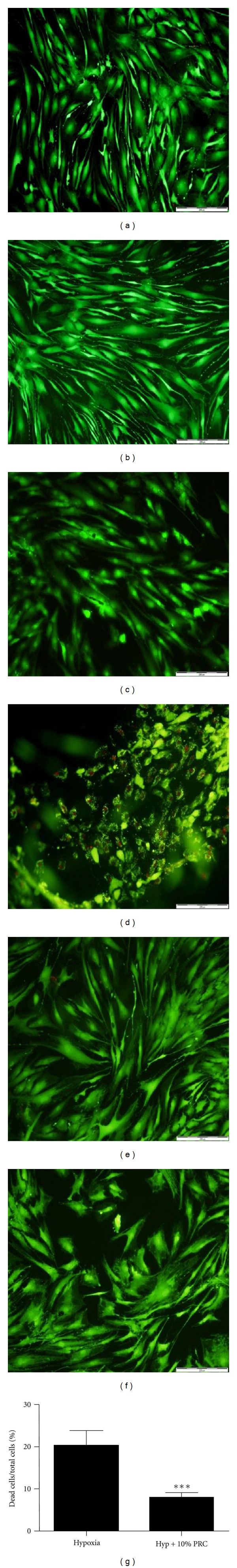
Platelet rich concentrate protects tenocytes against hypoxia-induced cell death. Live & Dead staining of tenocytes following treatment of cells for 48 h under normoxia alone (a), with the addition of 30 ug/mL insulin (b) or 10% PRC conditioned medium (c) and following 48 h with 1% oxygen (d), with addition of 30 ug/mL insulin (e) or 10% PRC conditioned medium (f). (g) Percentage of dead cells versus total cells with hypoxia treatment alone or in combination with 10% PRC (mean ± SD, *n* = 5). ***P = 0.0007 versus hypoxia.

Since insulin is not feasible for use as an *in vivo* therapy for tendon we tested platelet-rich plasma (PRP) as a source of growth factors. Culture medium conditioned overnight with 10% PRP was applied to tenocytes prior to hypoxic exposure for 48 h as above. Live & Dead staining showed no detectable cell death in normoxic cultures (Figures 7(a), 7(b), and 7(c)) with or without insulin (Figure 7(b)) or PRP (Figure 7(c)). Following 48 h of hypoxia the ratio of dead to live cells was clearly increased (Figure 7(d)) and treatment with either insulin (Figure 7(e)) or PRP (Figure 7(f)) provided protection. PRP reduced the percentage of dead cells from 20% to 8% of total cells (Figure 7(g)). 

## 4. Discussion

The biology of mature tenocytes differs from bone and skin fibroblasts, having more in common metabolically with cartilage. Tenocytes are capable of withstanding extreme mechanical conditions and reduced perfusion during sustained periods of loading. It is unclear whether tenocytes respond to low oxygen using the classical hypoxia response pathways or how vulnerable they are to acute hypoxic insult. We and others have noted a severe loss of cellularity in both ruptured and degenerating tendons and have recently described upregulation of HIF-1*α* and the hypoxia-induced death gene Bnip3, correlating with increased apoptosis in ruptured rotator cuff tendon [8, 9]. A clear understanding of the forces driving this loss of cells would allow development of effective protective strategies to enhance repair and recovery after trauma and also be of likely benefit during tendon graft integration.

We find that healthy human primary tenocytes respond to oxygen deprivation by rapidly upregulating protein levels of the classical hypoxia responsive transcription factor HIF1*α*. This is followed within 48 h by significant apoptosis. Two hypoxia-associated proapoptotic proteins, Bnip3, and Nix were upregulated in response to hypoxia, with Bnip3 levels peaking 4-fold higher then normoxic control between 8–16 h. Both proteins have been described as HIF-1*α* driven and their promoters contain hypoxia response elements [35]. Hypoxia did not downregulate the prosurvival proteins Bcl2 and Bcl-xL, nor was there any clear effect on other hypoxia-relevant proapoptotic proteins Bim, Bak, Bax, Puma, or Bad. Bmf and Noxa were not detectable in these tenocytes.

VEGF is an essential growth factor in the response of most tissues to traumatic injury involving disrupted blood supply. While VEGF induction has been reported *in vivo* following tendon rupture it has not been clear whether tenocytes themselves are mediating this in response to hypoxia, or other neighbouring cells including blood vessels. Also, induction by injury may be due to factors other than hypoxia since VEGF is also induced by proinflammatory cytokines and reactive oxygen species. We find that healthy human tenocytes express several isoforms of VEGF, including 4 splice variants of VEGF A as well as VEGF B and VEGF C. VEGF A is upregulated 8-fold at the mRNA level following hypoxia under both low (1% FCS) and high (10% FCS) serum conditions. Additionally, VEGF protein release into the culture medium is increased 4-fold by anoxia. This suggests that tenocytes display the typical mechanism by which hypoxic tissue attracts ingrowing blood vessels to restore normal perfusion following disruption due to injury. 

Although VEGF induction is necessary for tissue repair, tendon pathology has also been associated with sustained elevation of VEGF [3, 36]. This failure to resolve after healing is thought to be pathological and is currently being targeted using sclerosing drugs [3]. The outcome of VEGF elevation in rotator cuff stages is not known but our observation is that VEGF levels are lower than controls in the end-stage disease with large and massive tears [6]. Our data from healthy hamstring tenocytes suggests that VEGF elevation is a rapid and strong response to hypoxic insult, typical of most tissues. In the clinical setting chronic VEGF elevation once perfusion has been restored may be due to other factors such as inflammation and it will be important to study the contribution of acute versus chronic VEGF in tendon healing.

We found insulin and the growth factor cocktail released by PRP to be strongly antiapoptotic in hypoxic human hamstring tenocytes. Insulin dropped apoptosis levels to 50% and PRP to 40% of hypoxia induced levels in low serum conditions. In contrast VEGF or the commonly protective stable cAMP analogue cpt-cAMP were ineffective. This suggests that although VEGF is widely expressed in damaged tendons it is most likely of functional importance to other cell types such as endothelial cells. The protective effect of insulin was not due to prevention of upregulation of the hypoxia-induced transcription factors HIF1*α* or FOXO3A. However, both of these transcription factors display complex regulation. HIF1*α* stability and DNA-binding can be modified by multiple mechanisms [37]. In healthy or growth factor-stimulated cells FOXO3A is phosphorylated by PKB, causing its nuclear export and tethering to 14-3-3 adaptor protein in the cytoplasm [38]. Forkheads accumulate in the nucleus in response to JNK activation and when PKB activity drops [38]. The localization and DNA-binding activities of both HIF1*α* and FOXO3A should therefore be studied following insulin and hypoxia treatment of healthy tenocytes to better understand the protective mechanism.

In summary, normal human tenocytes respond to complete oxygen deprivation by activating typical response pathways. HIF1*α* and its target genes Bnip3 and VEGF are strongly upregulated and the damage response forkhead FOXO3A is also activated. Cell response was strongly modulated by the concentration of FCS, with reduced signaling under low serum conditions but considerably more apoptosis. This highlights the importance of perfusion and growth factors for the normal function of tendon, and the potential for increased susceptibility to cell death when tissue is disrupted by injury. Both insulin and PRP were effective in reducing hypoxia-induced cell death in cultured tenocytes, at least in the short term. PRP is highly practical as a rich source of autologous growth factors, since it can be extracted in theatre and reinjected into the same patient during repair or graft procedure. It is possible that application of these agents in the clinical setting may minimize hypoxia-induced damage to tendons in the clinical setting; however, future studies are indicated to more clearly define their role and efficacy clinically.
